# Genetic diversity and potential routes of transmission of *Mycobacterium bovis* in Mozambique

**DOI:** 10.1371/journal.pntd.0006147

**Published:** 2018-01-18

**Authors:** Adelina Machado, Teresa Rito, Solomon Ghebremichael, Nuelma Muhate, Gabriel Maxhuza, Custodia Macuamule, Ivania Moiane, Baltazar Macucule, Angelica Suzana Marranangumbe, Jorge Baptista, Joaquim Manguele, Tuija Koivula, Elizabeth Maria Streicher, Robin Mark Warren, Gunilla Kallenius, Paul van Helden, Margarida Correia-Neves

**Affiliations:** 1 Veterinary Faculty, Eduardo Mondlane University, Maputo, Mozambique; 2 DST-NRF Centre of Excellence for Biomedical Tuberculosis Research/SAMRC Centre for Tuberculosis Research, Division of Molecular Biology and Human Genetics, Faculty of Medicine and Health Sciences, Stellenbosch University, Tygerberg, Western Cape, South Africa; 3 Life and Health Sciences Research Institute (ICVS), School of Medicine, University of Minho, Braga, Portugal; 4 ICVS/3B's, PT Government Associate Laboratory, Braga/Guimarães, Portugal; 5 Department of Microbiology, Public Health Agency of Sweden, Solna, Sweden; 6 National Directorate for Veterinary Services, Ministry of Agriculture, Maputo, Mozambique; 7 Central Veterinary Laboratory, National Institute of Agriculture Research, Ministry of Agriculture, Maputo, Mozambique; 8 Department of Clinical Science and Education, Södersjukhuset, Karolinska Institutet, Stockholm, Sweden; University of California San Diego School of Medicine, UNITED STATES

## Abstract

Bovine tuberculosis is a zoonotic disease with largely unknown impact in Africa, with risk factors such as HIV and direct contact with animals or consumption of *Mycobacterium bovis* infected animal products. In order to understand and quantify this risk and design intervention strategies, good epidemiological studies are needed. Such studies can include molecular typing of *M*. *bovis* isolates. The aim of this study was to apply these tools to provide novel information concerning the distribution of bovine tuberculosis in cattle in Mozambique and thereby provide relevant information to guide policy development and strategies to contain the disease in livestock, and reduce the risk associated with transmission to humans. A collection of 178 *M*. *bovis* isolates was obtained from cattle in Mozambique. Using spoligotyping and regions of difference analysis, we classified the isolates into clonal complexes, thus reporting the first characterisation of *M*. *bovis* strains in this region. Data from MIRU-VNTR typing was used to compare isolates from a number of African countries, revealing a deeply geographically structured diversity of *M*. *bovis*. Eastern Africa appears to show high diversity, suggesting deep evolution in that region. The diversity of *M*. *bovis* in Africa does not seem to be a function of recent importation of animals, but is probably maintained within each particular region by constant reinfection from reservoir animals. Understanding the transmission routes of *M*. *bovis* in Mozambique and elsewhere is essential in order to focus public health and veterinary resources to contain bovine tuberculosis.

## Author summary

Bovine tuberculosis is a rather neglected zoonotic disease caused by *Mycobacterium bovis* that is of global concern owing to the persistence of the bacillus in reservoirs that can spread bovine tuberculosis between animals and humans. Africa remains understudied regarding this pathogen, and should be an area of concern given that in many regions the consumption of raw milk or meat from infected animals persists and the presence of HIV infection renders the population more susceptible. In order to control the disease, we need to understand *M*. *bovis* epidemiology, which includes the sources of infection. The important conclusion drawn from the work presented here is that there is a strong association between *M*. *bovis* genetic characteristics and geography. This implies that the diversity of *M*. *bovis* isolates in Mozambique does not seem to be caused by recent introductions to the territory, but is probably maintained within reservoirs in each particular region.

## Introduction

Bovine tuberculosis (BTB) is an infectious disease caused by *Mycobacterium bovis* that affects cattle, other domesticated animals and many free ranging or captive wildlife species. BTB is of global concern on at least three socio-economic levels: the negative impact on animal production; the potential spread to wildlife species; and the risk of zoonotic tuberculosis in humans [1].

BTB has a worldwide distribution with very low prevalence in most industrialized countries, although eradication has been claimed for a few countries only. Factors such as poor or no BTB veterinary control, consumption of uninspected raw meat and/or milk, difficult access to medical care, high prevalence of HIV/AIDS and malnutrition contribute to the increased risk for exposure and susceptibility of humans to *M*. *bovis* [2–4]. The maintenance of the pathogen in symptomatic and asymptomatic animals and in the environment creates conditions for dissemination not just to humans but also to a wide variety of wildlife species including endangered ones with the obvious negative consequences on conservation and tourism [5].

While BTB is known to be widespread in Africa, limited or outdated information exists concerning its precise distribution and incidence. In 1993, the World Health Organization (WHO), with the participation of the Food and Agriculture Organization (FAO), convened a meeting on BTB, where the worldwide public health significance of *M*. *bovis* in humans and animals was discussed. It was then concluded that data collected from most African countries, mainly from sub-Saharan Africa, were insufficient to reveal the true epidemiological picture of the disease, and it was recommended that collection of data on BTB should be prioritized [6].

Several small studies have been done, but the lack of detailed information in the vast majority of African countries is still of concern [1], particularly since the burden of BTB might be considerably underestimated in humans [7]. There are estimates suggesting that approximately 85% of cattle and 82% of the human population of Africa live in areas where BTB is either not controlled or only partially controlled [8]. This reflects the situation in Mozambique where efforts to improve BTB control are gradually being put into place. Implementation of a test and slaughter policy for cattle for the entire country would be the ideal strategy, but it is unrealistic at present, given resource limitations. Efforts to determine to what extent human tuberculosis cases in Mozambique are caused by *M*. *bovis* are ongoing [9]. By reviewing relevant scientific literature and public health reports, Müller and colleagues estimated the incidence of *M*. *bovis*, to be a median of 2.8% (range 0 to 37.7%) of total human tuberculosis cases in Africa [1], however more robust estimates are needed. According to WHO, over 70 thousand cases of tuberculosis in Africa in 2015 were caused by *M*. *bovis* [10], however, the absence of routine reports in the majority of countries imply considerable uncertainty for this estimate.

Scattered information from the past and results from ongoing studies in specific regions show that the prevalence of BTB varies widely from region to region and within each region. For example in the Govuro District, in the Southeast of Mozambique, 39.6% of cattle were skin test positive reactors for BTB [11]; while the disease was practically absent (0.98%) in cattle in the Limpopo National Park in the South West of Mozambique [12]. Although it is known that the differences in the BTB prevalence are associated with several risk factors, recent results from Govuro District clearly show that control of animal movement appears to be of high importance [11]. In that study, a high prevalence of BTB was observed in almost all livestock areas where small scale farming was practiced, in sharp contrast to that observed in the commercial sector, where no skin test positive animals were detected. While in the commercial sector animals are normally tested for BTB and kept in quarantine before being introduced, trading of animals among small-scale farmers is done without previous information concerning the BTB status of the animals. It is also in these familiar settings that close contacts with animals still occur, particularly for young children who manage daily livestock herding. In addition, due to their stature, children who herd animals may be highly exposed to *M*. *bovis* airborne transmission from infected animals [11].

It is increasingly clear that genotyping of *M*. *bovis* strains is a useful tool to identify possible transmission routes of BTB, and that the genotype of *M*. *bovis* isolates is largely dependent on the geographical area. Genotyping tools have already been widely used in Africa [13–25] but not in Mozambique.

We therefore obtained 178 *M*. *bovis* isolates from 8 Mozambican provinces and genotyped them using spoligotyping in the entire sample set and MIRU-VNTR and RD deletion testing in a subset. Additionally, we collected a comparative dataset from other African countries (based on spoligotyping patterns and MIRU-VNTRs) that allowed us to place the Mozambican strains in the African context. The analyses allowed us to address the following questions: is there a specific Mozambican *M*. *bovis* genetic pattern; do the *M*. *bovis* strains reveal cattle importation from neighbouring countries; is there evidence of movement of particular strains across districts within Mozambique; what is the position of Mozambique in the African scenario concerning *M*. *bovis*?

## Methods

### Samples

We obtained *M*. *bovis* isolates from 2007 to 2013, from samples collected during a BTB prevalence study (n = 228) and from samples sent to the Central Veterinary Laboratory in Maputo by district veterinary officers and farmers that suspected BTB at *post mortem*, either in the process of standard meat inspection or necropsy (n = 220) (Table 1). The samples from the prevalence study were from both small-scale and commercial herds of 10 selected districts, from the Provinces in the South (Manhiça, Magude, Chibuto and Govuro), Centre (Buzi, Mutarara and Gondola) and North (Mechanelas, Mogovolas and Angoche) of Mozambique. There is an overrepresentation of samples from the South due to a pilot study using test and slaughter performed in one district (Govuro), on two commercial farms that volunteer to cull the majority of the positive animals and send samples to the Central Veterinary Laboratory that is located also in the South.

**Table 1:** Sources of samples and number of isolates obtained.

Source of samples			Total samples obtained	Samples discarded	Negative samples	Positive samples	*M*.*tuberculosis* complex Positive	Isolates with spoligotype result
**Prevalence study**		**Tissue**	187	15	74	98	90	86
		**Milk**	41	8	16	17	11	9
**Laboratory samples**			220	27	89	104	84	75
**Other**								8
								178

A list of all herds from the respective districts was supplied by the provincial Veterinary Services department. A herd was defined as the group of animals from a commercial farm or a combination of animals owned by small-scale farmers sharing a dip tank or crush for regular veterinary assistance. Three localities per district were randomly selected. In commercial farms with fewer than one hundred animals the whole herd was tested. In commercial herds with more than one hundred animals, a random sample of at least one hundred animals older than 6 months was selected. High prevalence of BTB cases based on previous skin testing results together with good logistic conditions for the accomplishment of the work were the prime factors for district selection. Based on a strong response to *M*. *bovis* tuberculin purified protein derivative (PPD), animals were purchased and slaughtered. In two commercial farms (in Manhiça and Manica provinces), where skin test reactor cows could not be slaughtered, milk was retrieved and tested for the presence of *M*. *bovis* by microbiological culture.

Institutional permission to conduct the study was obtained from the National Directorate of Veterinary Services in Maputo, Mozambique (Nota 162/ MINAG/DNSV/900/2013) and the AUC (Animal Use Committee) of Stellenbosch University (SU-ACUM13-00009). Sampling and culling was performed as part of the Veterinary Services regular activity for disease control, following the procedures determined by the Mozambican Animal Health Regulation. The slaughter was done in registered abattoirs according to stipulated procedures. All mycobacterial cultures were performed in the National Tuberculosis Reference Laboratory, Ministry of Health Mozambique.

Sample data, including the name of the owner and the origin (province and district) of the animals were recorded when available. In Mozambique, for commercial beef production or small-scale farming, the animals are mostly from the local breed Landim and Brahman or crossbreeds between the two. Eight isolates were supplied by another study, four of which were from a milk production farm with Holstein Friesian and Jersey breeds.

### Tissue and milk processing and microbiological culture

Maceration of tissue samples was performed in a *stomacher* apparatus (in duplicate sterile stomacher bags with sterile distilled water). Samples were next decontaminated by adding 4% sodium hydroxide to the same volume of the macerate for 20 min. Supernatant was discarded after centrifugation at 3000 rpm for 20 min. For milk samples, equal volumes of milk and 4% sodium hydroxide were mixed and after 20 min they were centrifuged at 3000 rpm for 20 min. Distilled water was added to the sediment and after agitation and re-centrifugation the sediment obtained was used for inoculation of duplicate tubes with Löwenstein–Jensen medium with glycerol or with pyruvate. Incubation was done at 37 °C for up to 12 weeks. Fifty samples were discarded owing to contamination (Table 1). Isolates were identified as acid-fast bacilli with Ziehl–Neelsen staining (positive samples in Table 1). *M*. *tuberculosis* complex (MTC) was confirmed by PCR [26]. Briefly, DNA was extracted using a standardized protocol [27] and PCR amplified with the primers TB1-F 5’-GAA CAA TCC GGA GTT GAC AA-3’ and TB1-R 5’-AGC ACG CTG TCA ATC ATG TA-3’. The PCR protocol started with an initial denaturation step of 95 °C for 10 min, followed by 35 cycles of 95 °C for 1 min, 61 °C for 30 s and 72 °C for 2 min ending with a final step of 72 °C for 10 min. The PCR products were analysed using 1.5% agarose gels. All isolates that generated a product of around 370 bp were considered as belonging to the MTC. *M*. *bovis* was identified by spoligotyping [28] in 170 out of 185 MTC positive isolates tested (Table 1).

### Genotyping

Genotyping was performed using spoligotyping, region of difference (RD) analysis followed by MIRU-VNTR typing in a subset of the samples.

**Spoligotyping** was done on 178 isolates as described by Kamerbeek and colleagues [28], using membranes and equipment provided with a kit (Isogen Life Science B.V., Utrecht, The Netherlands, no longer available from this company). This method determines the presence or absence of 43 direct and variable repeat sequences within the direct repeat region, thereby generating spoligotype signatures which are hypothetically characteristic of defined strains [29].

**RD analysis** was based on PCR genomic analysis to determine the presence or absence of specific regions of difference (RD). It was done on 54 isolates by the assessment of the status of the RD Eu1, RD Af1 and RD Af2 regions. PCR products were visualized after electrophoresis on 1% agarose gels. RD Eu1 was amplified with primers Eu1_FW (5’-CCGATGAACTTGGCCCACAG-3’) and Eu1_Rv (5’–CGTGGTGGTGGGATGTCTTG-3’). A 1206 bp fragment was generated if the RD Eu1 was intact and a 400 bp fragment if the region was deleted [13,14,30]. For RD Af1 two primers targeting the flanking regions of RD Af1 (FW, 5’-ACTGGACCGGC AACGACCTGG-3’, and Rev, 5’-CGGGTGACCGTGAACTGCGAC-3’) and one primer hybridizing with the internal region of RDAf1 (Int Rev, 5’-CGGATCGCGGTGATCGTCGA-3’) were used [13,14] for a band of either 349 bp (intact) or 531 (deleted). For RD Af2 two primers targeting the flanking regions of RD Af2 (RD Af2_Fw, 5’-ACCGCCCTGTCCTATGTGAG -3’, RD Af2_Rev, 5’-TGACGGTTGCCTTTCTTGAC-3’) and one primer hybridizing with the internal region of RD Af2 (RD Af2_IntRev, 5’-CACTGTCTCCGCTCATCATG-3’) were used with bands of 458 bp (intact) and 707 bp (deleted) [14].

**MIRU-VNTR typing** was done on 59 strains using a standardized 24-locus MIRU-VNTR typing procedure [31]. The analysis was done using the MIRU-VNTR typing kit (Genoscreen, Lille, France). The PCR-products were run with 1200 LIZ size standard (GeneScan, Applied Biosystems) on ABI3500 sequencers. Sizing of the PCR-fragments and assignments of MIRU-VNTR alleles were done with the GeneMapper software version 4.1 (Applied Biosystems) according to the manufacturers’ instructions, generating a numerical profile for each strain. The genotyping of three extra VNTR markers (MIRU3232, MIRU3336 and MIRU2163a) was done by Genoscreen, France.

### Statistical and phylogenetic analysis

In order to evaluate relatedness of the spoligotype and VNTR patterns of the different samples we generated a dendogram using the UPGMA algorithm using the tool provided on the site http://www.miru-vntrplus.org/. The spoligotype patterns of the respective isolates were entered into the *Mbovis*.*org* database. In this database each unique spoligotype pattern is named by 'SB' followed by a four integer number e.g. SB0120 [32]. To further evaluate the relatedness of the 178 spoligotypes and possible clusters within the data we generated a minimum spanning tree using the tool provided on the site http://www.MIRU-VNTRplus.org, and the individual allelic diversity was calculated for all 24 MIRU-VNTR loci using the same site. A maximum difference of 1 mutation within a Clonal Complex was considered for the definition of clusters.

The index of discrimination [33,34] was calculated to determine the overall discriminatory power of the spoligotyping and MIRU-VNTR typing techniques using a tool provided on the site http://insilico.ehu.es/mini_tools/discriminatory_power. The number of isolates assigned to each type (spoligotyping and MIRU-VNTR) was introduced in the formula provided.

The genotyping of the three extra VNTR markers for increasing the discrimination power of the analysis had the drawback that these three markers could not be used in the MIRU-VNTRplus website. A UPGMA tree was calculated in MEGA [35] following a calculation of genetic distances between genotypes using the software Arlequin [36].

Phylogenetic reconstruction was performed on a larger dataset in order to contextualize the Mozambican diversity to the overall *M*. *bovis* diversity in Africa. In order to do so we collected data from 959 *M*. *bovis* strains (575 from Central and Western Africa, 104 from Eastern Africa, 6 from North Africa and 274 strains from Southern Africa) [13,14,25,37–44]. The VNTR markers were very divergent between studies but 5 of these were present in most studies and for more than 90% of the samples (viz. MIRU2165, MIRU2461, MIRU577, MIRU580 and MIRU3192 or ETR-A, -B, -C, -D and -E). The set have been suggested to provide enough resolution in the African context [45]. To the data from these, we added the 43 spoligotyping markers. Sample selection was based on a strategy to maximize this resolution (43 spoligotyping markers and 5 VNTRs) and thus studies/samples lacking these markers were excluded. On that premise, all available African *M*. *bovis* data in the literature following these parameters was included, although some geographic areas are underrepresented (North Africa) compared to others (Central/Western Africa). Reconstruction was done using a matrix of these 48 markers and by applying the reduced median algorithm [46] followed by the median joining algorithm [47], both present at the network software (freely available at http://www.fluxus-engineering.com). The algorithm is able to reconstruct missing genotypes allowing some data to be included even if there are missing markers. However to keep this extrapolation to a minimum, only samples that missed a single VNTR marker were included. For comparison two *M*. *tuberculosis* samples and two *M*. *caprae* samples were extracted from the MIRU-VNTRplus database and included in the analysis.

## Results

### Source of the isolates

The 178 *M*. *bovis* isolates were obtained from samples that originated from 8 out of the 11 Mozambican provinces from the Southern (n = 113), Central (n = 47) and Northern (n = 18) parts of the country, covering 21 of the 128 districts of Mozambique. The isolates originated from 34 small-scale farms with 103 isolates (1 to 6 isolates per farm, an average of about 3 isolates per farm) and 10 commercial farms with 68 isolates (1 to 17 isolates per farm, an average of 7 isolates per farm) (Table 2). The type of farm was unknown for 7 isolates. Five of the commercial farms were located in the South, 4 in the centre and 1 in the North of Mozambique (Table 2).

**Table 2:** Sources of *Mycobacterium bovis* isolates.

Regions	Number of Districts	Number of farms		Number of isolates			
		Small-scale	Commercial	Small-scale	Commercial	Type of farm not identified	Total
**South**	14	24	5	69	38	6	113
**Centre**	5	10	4	25	21	1	47
**North**	2	0	1	9	9	0	18
** **	**21**	**34**	**10**	**103**	**68**	**7**	**178**

### Spoligotype patterns and clustering analysis

Among the 178 *M*. *bovis* isolates, 15 individual spoligotype patterns were identified (S1 Table). All isolates lacked spacers 3, 9, 16, and 39 to 43, which is the signature profile of the *M*. *bovis* BCG vaccine strain, the ancestral spoligotype pattern of *M*. *bovis* (SB0120) as defined by the international *M*. *bovis* spoligotype database [www.mbovis.org]). Seven patterns were previously registered in the Mbovis.org database while the other 8 patterns were incorporated into the database with SB numbers (SB2304 to SB2311). The most common spoligotype was SB0961, accounting for more than half (n = 109, 61.2%) of the isolates. Seventeen isolates (9.5%) had the “BCG-like” spoligotype profile, identical to the spoligotype of the vaccine *M*. *bovis* BCG strain. The most frequent spoligotype SB0961 together with 6 others (SB1272, SB2304, SB2307, SB2308, SB2309 and SB2310) were of the “BCG-like derived” type, lacking one or more spacers in relation to SB0120 and not Af1, Af2 or Eu1 typical spoligotype patterns. Of the total isolates, 39 had spoligotypes lacking spacer 11, which is the signature of the Eu1 clonal complex [30]. Of these, 29 had the spoligotype SB0140, which has spacer 6 and 8 to 12 absent. Isolates with some of the previously not described spoligotypes (3 isolates with pattern SB2305, 4 isolates with SB2306 and 1 with pattern SB2311) were included in these patterns without spacer 11. None of the isolates had spacer 21 missing, a marker for isolates of the Eu2 clonal complex. None of the isolates had spoligotypes specific to the Af2 clonal complex, lacking spacers 3–7 [14]. The discriminatory index using spoligotyping was 0.5909.

RD analyses were performed to identify *M*. *bovis* clonal complexes. The 11 tested isolates with the typical Eu1 spoligotype SB0140 had the Eu1 specific deletion [30]. A possible Af1 spoligotype pattern (absence of spacer 30) did not have the Af1 deletion, a result supported by the phylogenetic analysis (below) that placed this sample as part of the “BCG-like derived” pattern. Additionally 42 isolates with spoligotypes SB0120, SB0140, SB0961, SB2305, SB2308 and SB2310 were tested for the Af1 deletion, 23 spoligotypes with SB0120, SB0140, SB0961, SB2305, SB2308 and SB2310 [13,14] were tested for the Af2 deletion and 14 for the Eu1 deletion (SB0961, SB1099, SB2305 and SB2306) and no deletions were detected.

**Fig 1:**
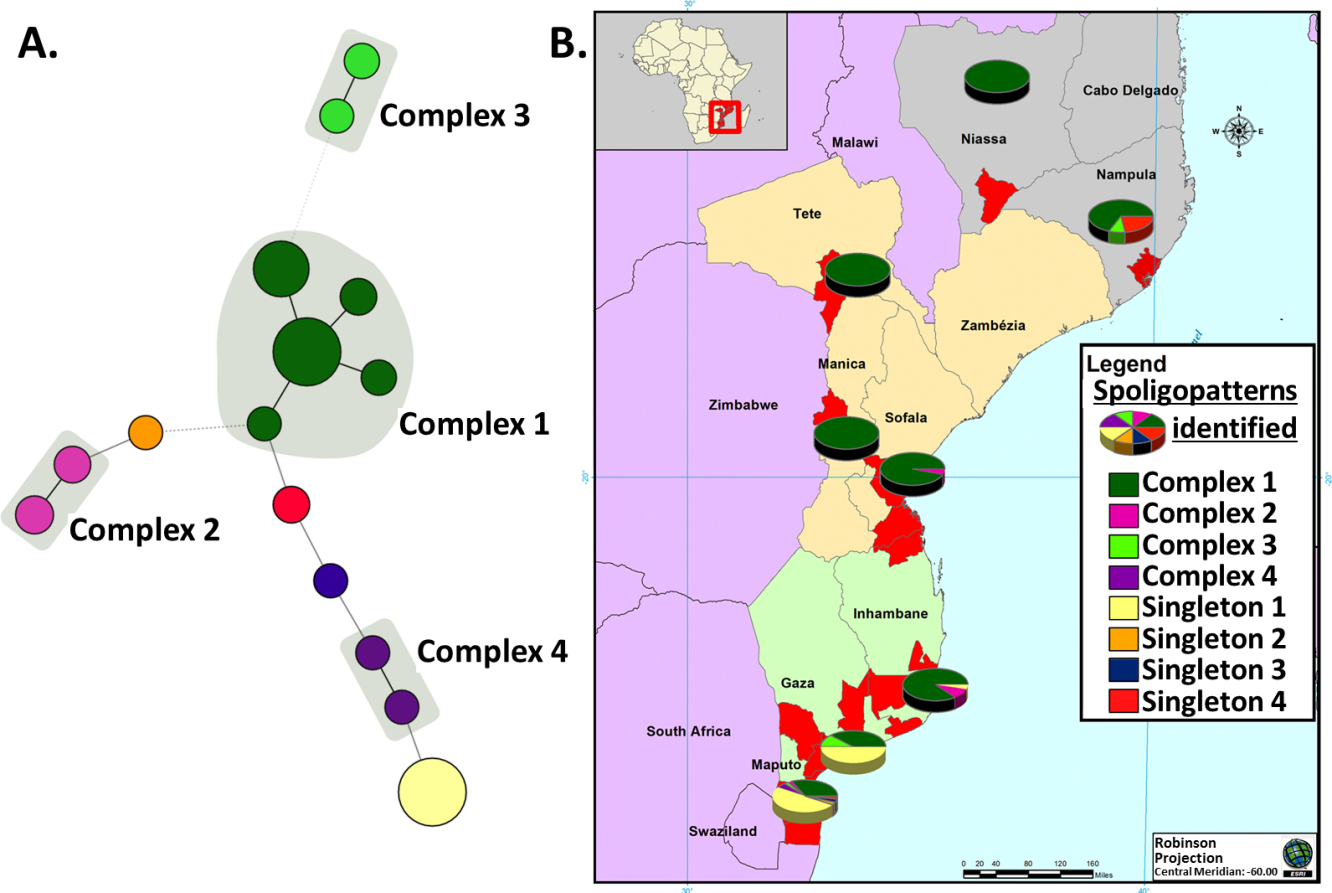
Clonal complexes and singletons identified in *Mycobacterium bovis* strains from Mozambique. **A. Results obtained from the calculation of the minimum spanning tree based on spoligotyping results.** In red a “BCG-like” spoligotype, in orange the “BCG-like derived” spoligotypes, in green the Af1 spoligotype and in blue the Eu1 spoligotypes. Singleton 1 corresponds to spoligopattern SB0140, singleton 2 to SB1272, singleton 3 to SB2311; singleton 4 to SB2304; complex 1 to SB0120, SB0961, SB1099, SB2307 and SB2309; complex 2 to SB2305 and SB2306; complex 3 to SB2308 and SB2310; complex 4 to SB0290 and SB2124. **B. Distribution of clonal complexes and singletons identified per province.** Pie charts represent the frequencies of the complex and singletons in the sampled provinces: Gaza, Inhambane, Manica, Maputo, Nampula, Niassa, Sofala and Tete. The map of Mozambique was created specifically for the manuscript using the licensed software ArcView—ESRI ArcMap 10.0 (Build 2414).

With calculation of the minimum spanning tree based on the spoligotyping results, 4 clonal complexes and 4 singletons (genotypes that are not clustered with other genotypes in the analysis) were identified (Fig 1A). Complexes 1 and 3 consisted of spoligotypes with “BCG-like derived” signatures, complex 1 also included the patterns with the “BCG-like” signature, while complexes 2 and 4 included spoligotypes with Eu1 signature.

### Geographical distribution of *M*. *bovis* spoligotypes

The *M*. *bovis* clonal complex distribution (based on spoligotyping results) according to Mozambican provinces is illustrated in Fig 1B. The most common spoligotype, SB0961, was detected in all of the provinces investigated. Similarly, isolates with the spoligotype SB0120 were present in most provinces, in both commercial and small-scale farms. The 29 isolates with the spoligotype SB0140, likely from the Eu1 clonal complex, were obtained from animals from the South of Mozambique only, [Maputo (n = 22), Inhambane (n = 2) and Gaza (n = 5)]. Of these 29 isolates, 23 were from 3 commercial farms, 4 have unknown origin (1 from Inhambane, 1 from Maputo and 2 from Gaza provinces) and 2 from two different small-scale farms (from Inhambane province and from Maputo province). The 10 isolates with Eu1 signature other than SB0140 were also all from the south of Mozambique, 4 from commercial farms (SB0290, SB2124, and SB2311) and 6 from small-scale farms in Maputo and Inhambane, except one (SB2305), that was isolated from Sofala province (Centre of Mozambique). Only one of the small-scale farms generated 6 isolates (5 isolates with pattern SB0961 and 1 with SB0120); the other yielded only 1 or 2 isolates that in some cases had identical patterns. In the commercial farms there was a variability of patterns, ranging from 1 to 6 different patterns per farm.

### Subtyping of spoligotypes with MIRU-VNTR analysis

**Fig 2:**
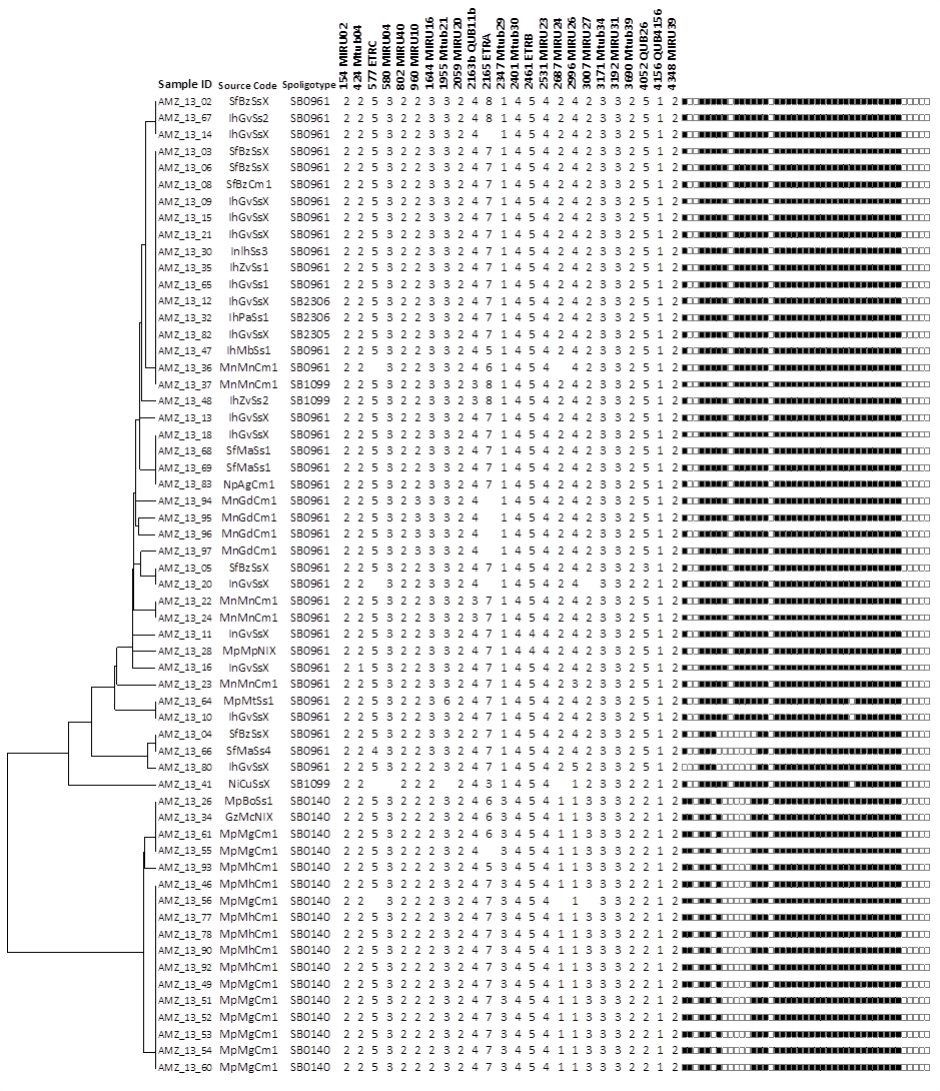
Dendogram displaying the MIRU-24 loci VNTR profiles of the 59 *Mycobacterium bovis* isolates evaluated. The figure indicates the sample code; the source of the samples (Xx00000 –province, 00Xx000 –district, 0000Xx–type of farm and 000000X –the farm: Mp–Maputo, Gz–Gaza, Ih–Inhambane, Sf- Sofala, Mn–Manica, Np–Nampula, Ni–Niassa; Bo–Boane, Mt–Matutuine, Mh–Manhiça, Mg–Magude, Mc–Macia, Zv–Zavala, Mb–Morrumbene, Pa–Panda, Gv–Govuro; Ma—Machanga, Bz–Buzi; Gd–Gondola, Mn–Manica, Ag–Angoche, Cu–Cumba; Cm–comercial, Ss–small-scale, NI—Not identified, X–Not known); the spoligopatterns; the multiple-locus variable-number tandem repeat analysis (MLVA) identified with the designation numbers obtained using the site http://www.MIRU-VNTRplus.org; and the MIRU-VNTR 24 loci code profiles.

To further discriminate the *M*. *bovis* isolates defined by spoligotyping and in an attempt to define potential specific links between farms (potentially associated to transmission or common source of infection), 24-locus MIRU-VNTR typing was done on 59 isolates. These isolates were selected based on the better preserved material from which bacteria and/or DNA remained. Amongst the isolates tested, we found a fairly high degree of diversity, which resulted in a splitting of the 59 isolates into 23 different MIRU-VNTR types (Fig 2). The loci MIRU154, MIRU580, MIRU802, MIRU2059, MIRU2401, MIRU2531, MIRU3171, MIRU3192, MIRU3690, MIRU4156 and MIRU4348 showed no variation in all the isolates analysed. From the loci that showed variability, MIRU2165 (ETR-A), MIRU2687 (MIRU24), MIRU2996 (MIRU26), MIRU4052 (QUB26), MIRU3007 (MIRU27) and MIRU2347 (Mtub29) showed higher variability, with allelic diversities ranging from 0.50 to 0.43. The discriminatory index using MIRU-VNTR was 0.8708. Thirty-six isolates with spoligotype SB0961 could be further differentiated into 15 new types, 17 isolates with SB0140 into 5 types and 3 isolates with SB1099 into 2 different MIRU-VNTR types.

The Multiple-locus variable-number tandem repeat analysis (MLVA) identified major clusters: “16919–270” shared by 11 isolates of the spoligopattern SB0140 from 2 different commercial farms located in 2 different districts (Manhiça and Magude) and “16911–1358” shared by 15 isolates with spoligopattern SB0961, 12 being from various small-scale farms and 1 from a commercial farm from 3 neighbouring districts (Machanga and Buzi from Sofala Province and Govuro from Inhambane province) and the remaining 2 from 2 different districts (Zavala and Angoche). Another cluster of interest was “16917–270” (SB0140) shared by 3 isolates all from different districts (Boane and Macia and Magude), 1 isolate was from a commercial farm, 1 from a small-scale farm and the third was of unknown origin.

The introduction of 3 extra VNTR markers (MIRU3232, MIRU3336 and MIRU2163a) resulted in minor changes only in the overall dendogram (S1 Fig), however with an increment of the discriminatory index to 0.9293.

### Contextualizing Mozambican *M*. *bovis* diversity with African countries

**Fig 3:**
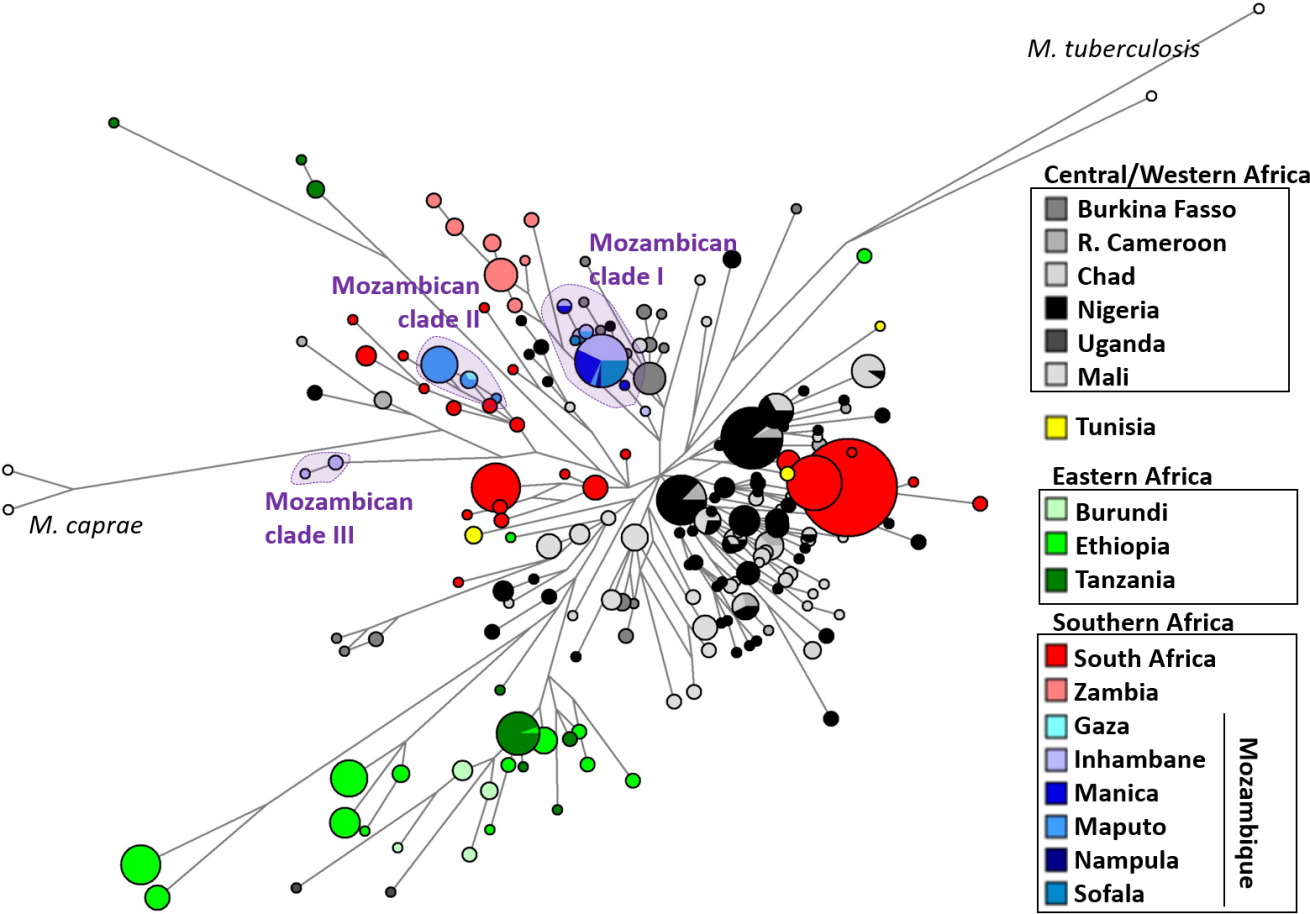
Median joining network of the diversity of *Mycobacterium bovis* in Africa based on the 43 spoligotype spacers and 5 VNTRs. Samples were coloured according to their geography. Figure was made using freely available phylogenetic software network (http://www.fluxus-engineering.com.

In order to integrate the Mozambican *M*. *bovis* data obtained here into the overall African diversity, we generated a general phylogenetic network based on spoligotyping data and 5 VNTRs collected from published manuscripts (S2 Table) [13,14,37–44]. Median networks, the approach presented here to establish the hypothetical links, attempts to establish evolutionary relationships between genotypes, such that nodes in the tree represent ancestral genotypes. The network displays a set of clades, geographically structured across the continent. The comparative genotypes of *M*. *tuberculosis* and *M*. *caprae* were clearly differentiated from the *M*. *bovis* strains (Fig 3). The analysis also allowed the inference of the positioning of Mozambican *M*. *bovis* diversity in the general African scenario. One important aspect that emerged from the analysis is that diversity seems deeply geographically structured with sharing of genotypes occurring mostly between neighbouring regions.

Mozambican genotypes fall into 3 main monophyletic clades (Fig 3). The first one, labelled clade I in Fig 3, is found across Mozambique and corresponds to the “BCG-like” and the “BCG-like derived” clades mentioned above. This clade is related to a minor branch from Cameroon, but shows deeper phylogenetic relationship to southern African isolates.

The second clade (clade II) is related to South African genotypes, including not only genotypes detected in cattle but also in wildlife, and it is only detected in Gaza and Maputo, the 2 southern sampling locations matching the geographical structuring. This clade corresponds to the Eu1-type complex mentioned above.

A third minor clade (clade III) with previously unknown spoligotypes SB2305 and SB2306, was detected only in Inhambane and does not appear to be closely related to any other genotypes in the available dataset.

## Discussion

This is the most extensive study on genetic diversity of *M*. *bovis* isolates from Mozambique. The genetic diversity of *M*. *bovis* was integrated in the general diversity of Africa, considering the publicly available strains, in what is the major strain comparative study of the continent to date.

Generally, we detected a few clusters that are most likely localised. The dominant clusters are from the “BCG-like” and “BCG-like derived” spoligotype patterns, which are widely spread in Mozambique and neighbouring Southern African countries, but so far absent in the remaining Sub-Saharan Africa [48].

In this study, 39 isolates had the signature of the Eu1 clonal complex. This group of strains is common in the British Isles, the New World, as well as UK’s former colonies and trading partners [30]. The global distribution of these strains suggests a complex history involving recent international cattle trade [48] since Eu1 is rare or absent in the African countries thus far surveyed, except for South Africa [17]. In South Africa, Eu1 strains (SB0140) were identified in samples from cattle [17] and from wildlife [37]. All except one (SB2305) of the 39 isolates with the Eu1 spoligotype pattern were isolated in the South of Mozambique, and the majority were from commercial farms (n = 26), which may have imported infected cattle from South Africa where the Eu1 clonal complex is common [30]. Information collected from the reports of the veterinary services showed that cattle importation to the South of Mozambique in the years 1950 to 1957 were mainly from South Africa (467 animals) and Portugal (59 animals) while in the centre of the country cattle were imported mainly from Zimbabwe (917 animals) but also from South Africa (116 animals). In the North, cattle were imported mainly from Malawi.

In the phylogenetic network, many Mozambican isolates represent sub-branches of the South African clades. The Mozambican strains could be placed into only 2 monophyletic clades (clades I and II in Fig 3), meaning at most two introductions of *M*. *bovis*, one for each clade.

A third minor cluster was identified, with spoligotype patterns SB2305 and SB2306, initially thought to be from the Eu1 clonal complex but lacking the RD Eu1 deletion. These samples formed an independent cluster in the network with no close relationship, possibly suggesting a local deep Mozambican clade. It is interesting to point out that Eastern Africa (Ethiopia, Burundi and Tanzania) seem to display very deep diversity in terms of *M*. *bovis* strains in our phylogenetic analysis, suggesting a deeper evolution of *M*. *bovis* in that region. These deeper clades likely correspond to the Af2 clonal complex that have been recovered from cattle in East Africa [14], Uganda, Burundi, Tanzania and Ethiopia. However, no isolates with the spoligotype marker of the Af2 clonal complex (absence of spacers 3 to 7) were identified in our sample. Although Tanzania borders Mozambique, our data suggest that cattle movement between the two countries is limited. However sample size from the North of Mozambique where there is a shared border with Tanzania, was very small and one needs to consider that this signal might be present at low prevalence and was not seen in this study.

The “BCG-like” strains are dominant in Mozambique, being present from North to South. These strains dominate in most of mainland Europe [48], but also in North Africa (e.g. Algeria [49]), indicating a probable importation of live cattle from Europe (mostly from France) to Algeria. In Zambia the “BCG-like” spoligotype is also dominant [50] with the predominant ancestral spoligotype in cattle [50,51] and wildlife [51]. Thus, *M*. *bovis* might have been introduced from Europe into the two neighbouring countries. The positioning of the Mozambican and Zambian branches in the network suggests two independent sources of introduction. The predominant spoligotyping pattern identified in our study, SB0961, was also isolated in Argentina [52], in the Czech Republic and Slovakia [53], but at very low frequencies. It is likely that the genotype was one of the first ones to be introduced in Mozambique, which became common over time. The phylogenetic network suggests that all members derive from a single ancestor.

BTB control needs to focus not just on animals but also on humans and their dynamics. Cattle are obviously the most direct source of BTB transmission between themselves and transmission to humans. In Mozambique the overall prevalence of BTB in cattle is 13.6% [11,12]. In terms of policies, given the current unrealistic likelihood of testing all animals in Mozambique, there is a need to reinforce the regulations that require a negative BTB test result before cattle importation. The same should be enforced for internal movements, since the frequency of shared genotypes from cattle originating from different parts of the country strongly suggests internal transmission of BTB. In that sense, looking at the geographic structuring of the African data in the phylogenetic reconstruction (Fig 3) it is likely that the major current issue in the continent is not spread of genotypes across borders but mainly the maintenance of existing local genotypes in the various countries or regions.

In addition to meat consumption of infected animals, one possible source of transmission to humans is milk. We found that analysis of a single milk sample was sufficient to obtain a *M*. *bovis* isolate from as many as 9 out of 41 (22%) skin test positive cows. Thus, milk that is frequently consumed with no treatment is on one hand a serious potential source of transmission to calves and humans, and on the other hand, it represents an easily available source of isolates from infected herds to estimate prevalence of BTB. *M*. *bovis* infection in humans has been clearly shown in several African countries, as is shown in the network (S2 Fig). The very limited studies done in Mozambique thus far have not shown BTB in humans, but studies are very limited and have not been done on the populations known to be at higher risk.

The relevance of the presence of *M*. *bovis* in wildlife is often overlooked. BTB has been reported in wildlife in Mozambique [54]. BTB in natural populations is very difficult to control, since it requires a different set of expertise (ecologists, zoologists, ethologists) demanding a deeper collaboration between very different entities. While BTB is a risk for natural populations, they could also represent a reservoir of *M*. *bovis* that can re-infect cattle populations [55]. Another issue is that wildlife borders are limited by ecological contexts and not political borders, meaning that although strict measures and surveillance on cattle importation might be in place, wildlife movement, particularly where border fences are dropped for transborder parks, cannot be controlled.

The HIV epidemic in Africa, affecting over 25.5 million individuals, places the population of this continent at higher risk of contracting BTB than elsewhere globally. The impact of BTB as a zoonotic disease in Mozambique remains unknown and no study addressed specifically the prevalence of BTB in risk groups, namely individuals with HIV. Given that BTB has been estimated to range from 0% to 37.7% of reported human TB cases in Africa [1], we conclude that there is a considerable and underestimated BTB risk to humans in Africa. This is particularly concerning given that the emergence of multidrug-resistant strains of *M*. *bovis* has been reported [56,57].

It is therefore of vital importance to continue the efforts made in Mozambique in order to completely characterise and understand the extent of BTB. The information concerning *M*. *bovis* presented here represents a foundation stone in that process.

## Supporting information

S1 Fig Dendogram displaying the genotypic profiles of the 59 *Mycobacterium bovis* isolates evaluated using 27 MIRU-VNTR markers. The figure indicates the sample code; the source code of the samples (Xx00000 –province, 00Xx000 –district, 0000Xx–type of farm and 000000X –the farm; Mp–Maputo, Gz–Gaza, Ih–Inhambane, Sf- Sofala, Mn–Manica, Np–Nampula, Ni–Niassa; Bo–Boane, Mt–Matutuine, Mh–Manhiça, Mg–Magude, Mc–Macia, Zv–Zavala, Mb–Morrumbene, Pa–Panda, Gv–Govuro; Ma—Machanga, Bz–Buzi; Gd–Gondola, Mn–Manica, Ag–Angoche, Cu–Cumba; Cm–comercial, Ss–small-scale, NI—Not identified, X–Not known); the spoligopatterns; and the MIRU-VNTR 24 loci code profiles with 3 additional typed markers: MIRU3232, MIRU3336 and MIRU2163a.
(TIF)

S2 Fig Median joining network of the diversity of *Mycobacterium bovis* in Africa based on the 43 sploligotype spacers and 5 MIRU-VNTRs. Samples are coloured according to the infected host.
(TIF)

S1 Table Summary of typing results per isolate and isolate sources. The table indicates Sample ID; Source code (Xx00000 –province, 00Xx000 –district, 0000Xx–type of farm and 000000X –the farm; Mp–Maputo, Gz–Gaza, Ih–Inhambane, Sf- Sofala, Mn–Manica, Tt–Tete, Np–Nampula, Ni–Niassa; Bo–Boane, Ct–Catuane, Mt–Matutuine, Mh–Manhiça, Mg–Magude, Mr–Marracuene, Nm–Namaacha, Cb–Chibuto, Mc–Macia, Zv–Zavala, Mb–Morrumbene, Pa–Panda, Gv–Govuro; Ma—Machanga, Bz–Buzi; Gd–Gondola, Mn–Manica, Ag–Angoche, Cu–Cumba; Ch- Changara; Cm–comercial, Ss–small-scale, NI—Not identified, X–Not known, ND–Not done, NR- No result); 43 number spoligotypes profiles; spoligopattern; source; district; province; species from where *M*. *bovis* was isolated; Regions of Difference (RD); MIRU Code; multiple-locus variable-number tandem repeat analysis (MLVA); Origin; Af1; Af2; Eu1.
(XLSX)

S2 Table Global African database of *Mycobacterium bovis* collected from the literature. The table indicates sample ID; reference from the work from where that data was collected; geographical code; African region; sub-region; species; collection date; MIRU-VNTR profile and spoligopatterns.
(XLSX)
