# Supplementary figures and images for: Genetic diversity and potential routes of transmission of Mycobacterium bovis in Mozambique
Source: PLoS Negl Trop Dis. 2018 Jan 18;12(1):e0006147. doi: 10.1371/journal.pntd.0006147 (PMC5772998; doi:10.1371/journal.pntd.0006147)

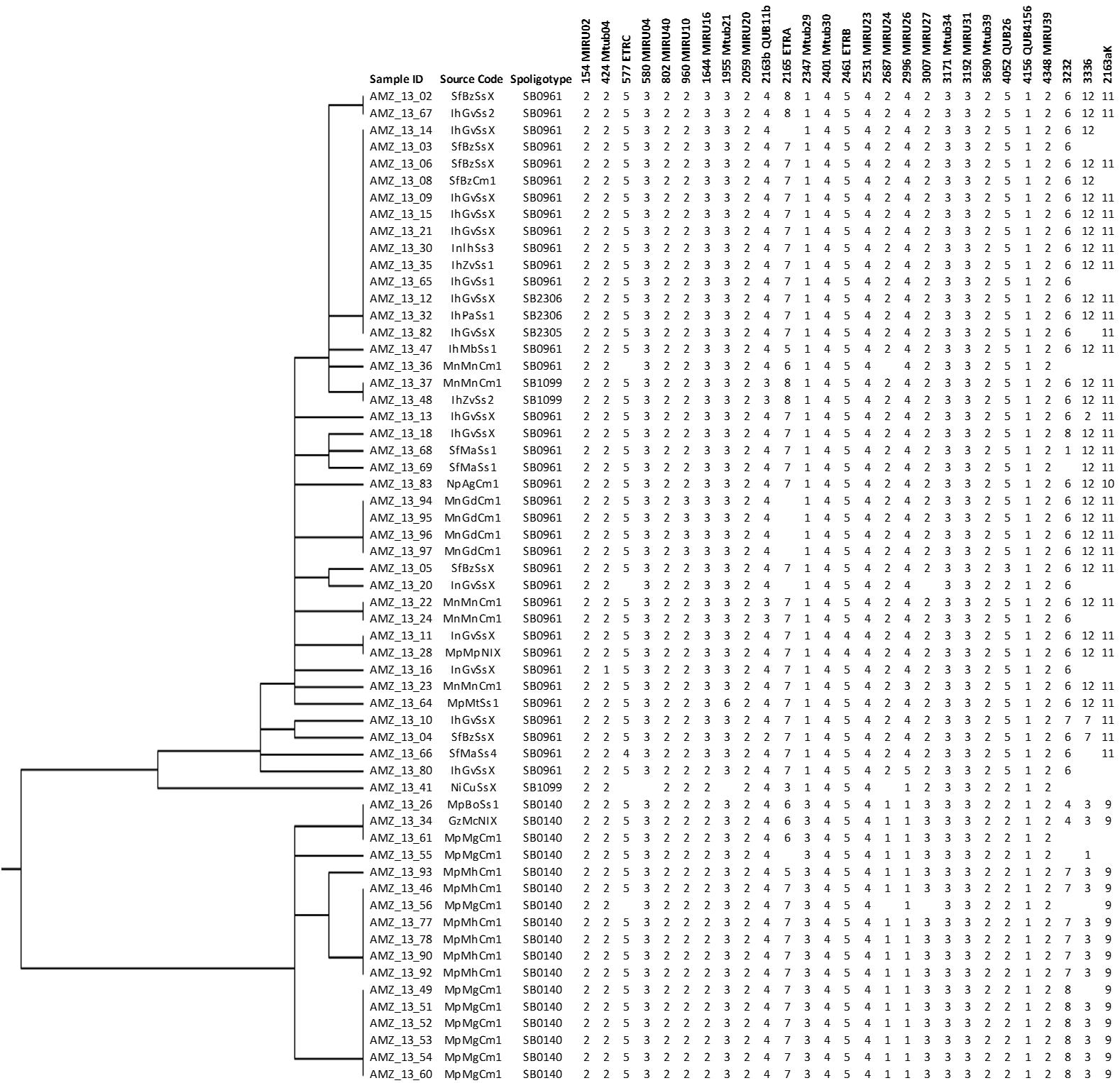

Supplement: S1 Fig — The figure indicates the sample code; the source code of the samples (Xx00000 –province, 00Xx000 –district, 0000Xx–type of farm and 000000X –the farm; Mp–Maputo, Gz–Gaza, Ih–Inhambane, Sf- Sofala, Mn–Manica, Np–Nampula, Ni–Niassa; Bo–Boane, Mt–Matutuine, Mh–Manhiça, Mg–Magude, Mc–Macia, Zv–Zavala, Mb–Morrumbene, Pa–Panda, Gv–Govuro; Ma—Machanga, Bz–Buzi; Gd–Gondola, Mn–Manica, Ag–Angoche, Cu–Cumba; Cm–comercial, Ss–small-scale, NI—Not identified, X–Not known); the spoligopatterns; and the MIRU-VNTR 24 loci code profiles with 3 additional typed markers: MIRU3232, MIRU3336 and MIRU2163a. (TIF) [file pntd.0006147.s001.tif]

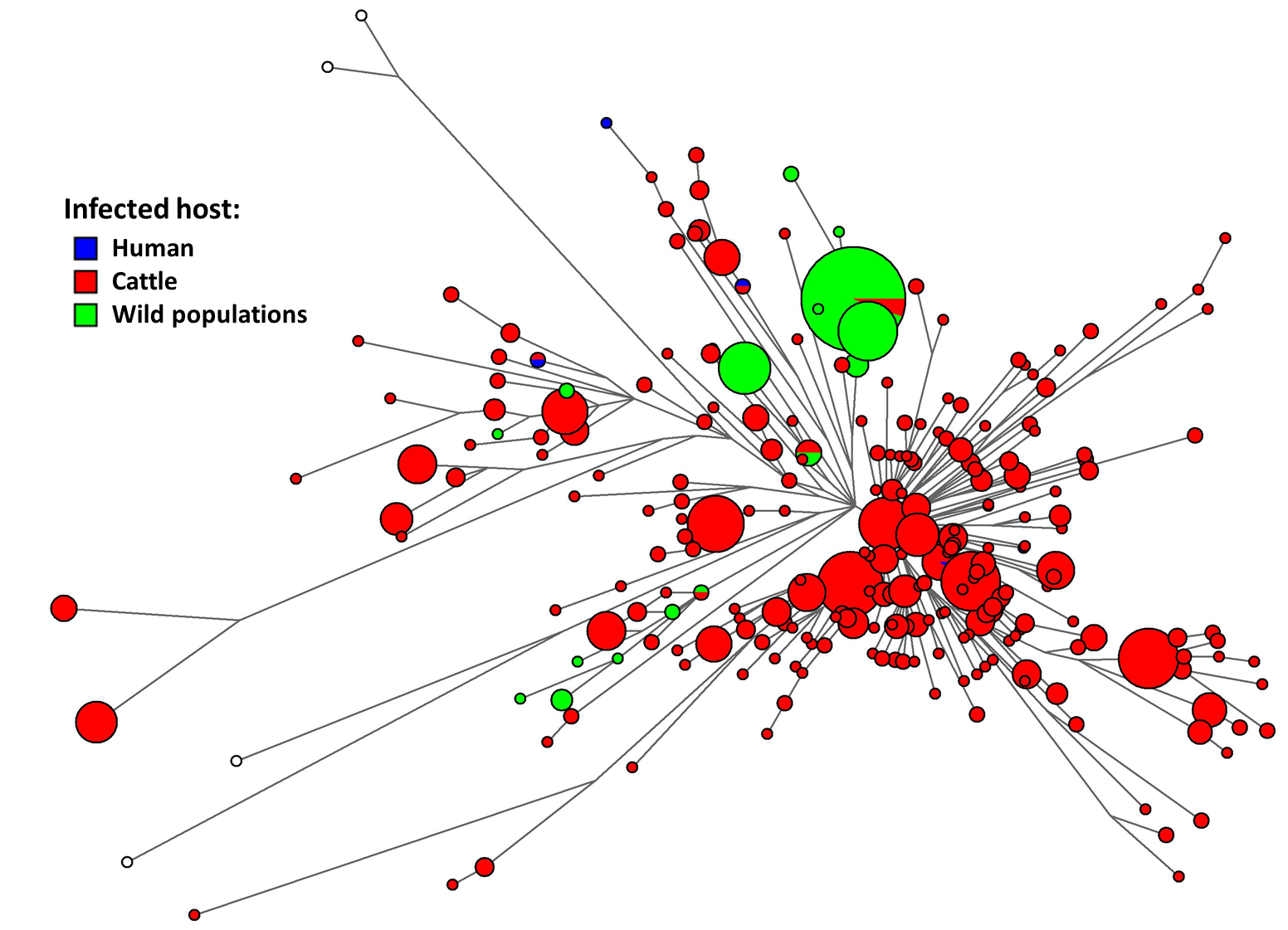

Supplement: S2 Fig — Samples are coloured according to the infected host. (TIF) [file pntd.0006147.s002.tif]
